# Familial systemic lupus erythematosus with pleural and articular involvement mimicking tuberculosis: a case report

**DOI:** 10.1097/MS9.0000000000004686

**Published:** 2026-01-21

**Authors:** Rajan Gyawali, Lokmani Bhandari, Sushant Shah, Sasisth Sah, Abhaya Acharya, Shailee Timsina, RItesh Kumar Shah

**Affiliations:** a Department of Internal Medicine, Tribhuwan University Teaching Hospital, Kathmandu, Nepal; b Maharajgunj Medical Campus, Institute of Medicine, Tribhuvan University, Kathmandu, Nepal; c Department of Internal Medicine, College of Medical Sciences, Kathmandu University, Bharatpur, Nepal; d Department of Internal Medicine, Tribhuvan University Institute of Medicine, Nepal

**Keywords:** autoimmune disease, familial SLE, pleuritis, polyarthritis, systemic lupus erythematosus

## Abstract

**Introduction:**

Systemic lupus erythematosus (SLE), a multisystemic disease with a global prevalence of 20-150/100 000, predominantly affecting women may have a familial clustering, suggesting a strong genetic predisposition and emphasizing the role of heritable factors in disease pathogenesis. In tuberculosis-endemic regions, pleural effusions are frequently misattributed to tuberculosis, leading to delays in the correct diagnosis and appropriate treatment.

**Case presentation:**

A 42-year-old female with a history of continuous chest pain, aggravated on inspiration, intermittent fever with night sweats, chills and rigor, long standing joint pain involving small joints of hand, knee, and elbow with swelling and elbow has a family history of SLE and was started on anti-tuberculosis therapy, which showed no improvement. Examination was suggestive of pleura involvement. Investigations showed strongly positive antinuclear antibody with a homogeneous pattern and mitotic spindle enhancement. The autoantibody profile showed positivity for anti-dsDNA, anti-Smith, U1-RNP, SSA (Ro), SSB (La), Ro-52, histone, and nucleosome antibodies. Rheumatoid factor was also positive, while KU antibody and DFS70 were borderline positive. Antimalarial drug, corticosteroid, and immune-suppressants with supportive management were given.

**Discussion:**

Familial SLE is uncommon but clearly delineated, with first-degree relatives at far greater risk. Misdiagnosis with tuberculosis is typical in endemic areas due to the overlap of symptoms like pleuritis and fever. Diagnosis can only be made early by a thorough immunologic workup and family history.

**Conclusion:**

This case highlights the importance of considering SLE in patients with unresolved pleural and joint symptoms, especially in the presence of a strong family history. Early recognition of familial SLE can avoid misdiagnosis and lead to better outcomes through timely initiation of immunosuppressive therapy.

## Introduction

Systemic lupus erythematosus (SLE) is a chronic, multisystem autoimmune disease characterized by the production of a variety of autoantibodies and diverse clinical manifestations affecting the skin, joints, kidneys, serosal membranes, hematologic system, and central nervous system^[1]^.The global prevalence of SLE varies between 20 and 150 cases per 100 000 individuals, with significant regional and ethnic differences. SLE predominantly affects women, particularly those of childbearing age, with a reported female-to-male ratio of approximately 9:1^[2]^.

**HIGHLIGHTS**

- A 42-year-old female presented with chronic chest pain, fever, and long-standing joint pain.
- She had a strong family history of SLE, with her mother and sibling also diagnosed.
- She was initially misdiagnosed with tuberculosis and received anti-tubercular therapy without improvement.
- Immunological studies showed a strongly positive antinuclear antibody and a wide range of positive autoantibodies, including anti-dsDNA and anti-Smith.
- The diagnosis of systemic lupus erythematosus with pleural involvement and polyarthritis was made based on clinical features, autoantibody profile, and family history.
- Treatment included corticosteroids, hydroxychloroquine, and azathioprine, along with supportive care.

Although familial clustering of SLE has been reported, it remains relatively rare, with only about 10%–12% of cases occurring in patients who have an affected first-degree relative. The presence of SLE in multiple first-degree relatives suggests a strong genetic predisposition and emphasizes the role of heritable factors in disease pathogenesis^[3]^. Serositis, including pleural and pericardial involvement, is a recognized but often underappreciated manifestation of SLE^[4]^. In tuberculosis-endemic regions, pleural effusions are frequently misattributed to tuberculosis, leading to delays in the correct diagnosis and appropriate treatment of underlying autoimmune conditions.

We present a rare case of a middle-aged female with longstanding inflammatory polyarthritis and chronic pleural symptoms who was initially misdiagnosed as having tuberculosis and empirically treated, without improvement. The patient had a strong family history of SLE involving both her mother and sibling, highlighting the importance of recognizing familial forms of SLE. This case underscores the need for a high index of suspicion for SLE in patients presenting with serositis and polyarthritis, even in tuberculosis-endemic settings, and emphasizes the significance of detailed immunological workup and family history in establishing an accurate diagnosis.

This case report was prepared in accordance with SCARE criteria^[5]^.

## Case presentation

A 42-year-old female from Kathmandu, Nepal, presented at Tribhuvan University Teaching Hospital, a tertiary care center with a history of dull, continuous chest pain for the past 8 months, which was aggravated by deep inspiration and associated with a sensation of heaviness. The pain was not accompanied by cough. She also reported intermittent fever over the same duration, mainly in the evenings, along with chills, rigors, and night sweats, all relieved by paracetamol. Over the last 3 months, she experienced progressive easy fatigability that significantly limited her daily activities, along with decreased appetite. She also had a long-standing history of 10 years of joint pain involving the small joints of the hands, elbows, and knees, with associated swelling and morning stiffness that improved with medication. There was no history of photosensitivity, malar rash, oral ulcers, hair loss, or Raynaud’s phenomenon. Notably, there was a positive family history of SLE in both her mother and sibling, suggesting a familial predisposition. The mother had a history of fever, jaundice, and arthritis, and after multiple investigations, she was diagnosed with SLE, whereas her sister presented with arthritis, malar rash, and oral ulcers and was diagnosed with SLE 3 months later. Three months ago, the patient was empirically started on anti-tubercular therapy (ATT) at a peripheral health care center before referral to our hospital. The decision to initiate ATT was based primarily on clinical suspicion of tuberculosis due to persistent pleuritic chest pain, fever, and night sweats in the setting of a presumed pleural effusion, without confirmatory microbiological or histopathological evidence. However there was no improvement in the patient condition. Imaging investigations revealed that Chest X-ray showed blunting of the right costophrenic angle, consistent with pleural effusion, but no parenchymal infiltrates or cavitary lesions were present. Electrocardiography (ECG) shows normal sinus rhythm, and on echocardiography, all the heart chambers look normal with an ejection fraction of 60%.

**Figure 1.**
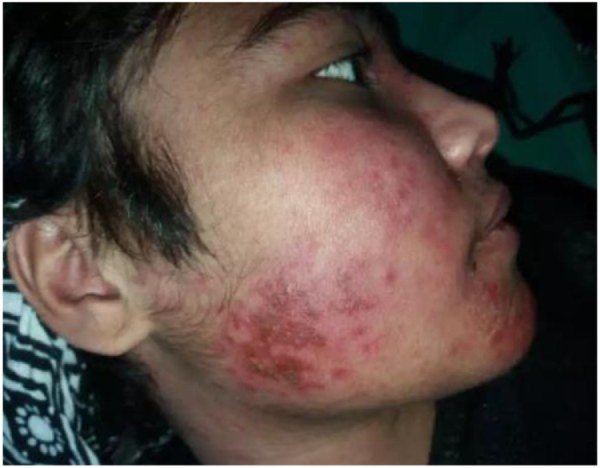
Erythematous plaques, pustules, and crusted lesions over the right cheek and jawline suggestive of cutaneous lupus erythematosus, possibly acute or discoid subtype.

**Figure 2.**
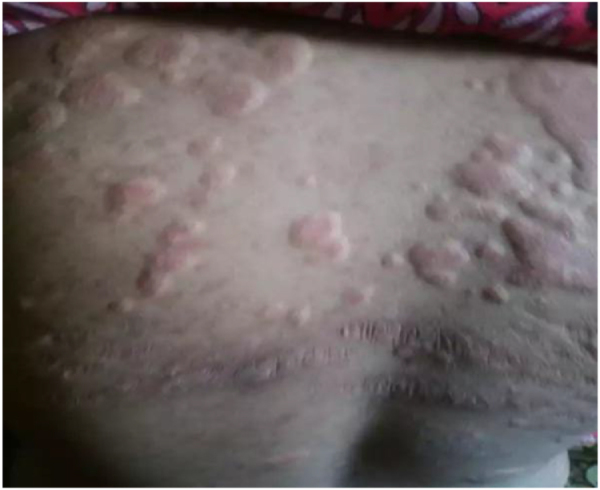
Multiple erythematous, scaly, and hyperpigmented plaques with lichenification over the back.

**Figure 3.**
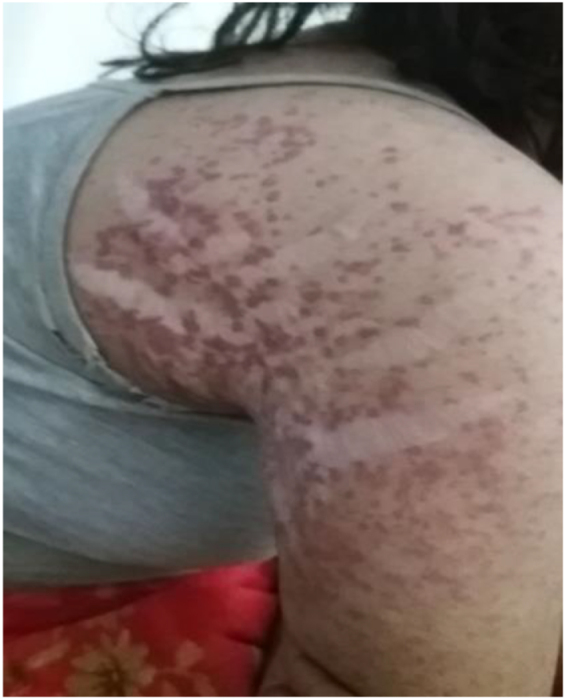
Rash with red-brown spots and lighter areas on the arm and shoulder.

**Figure 4.**
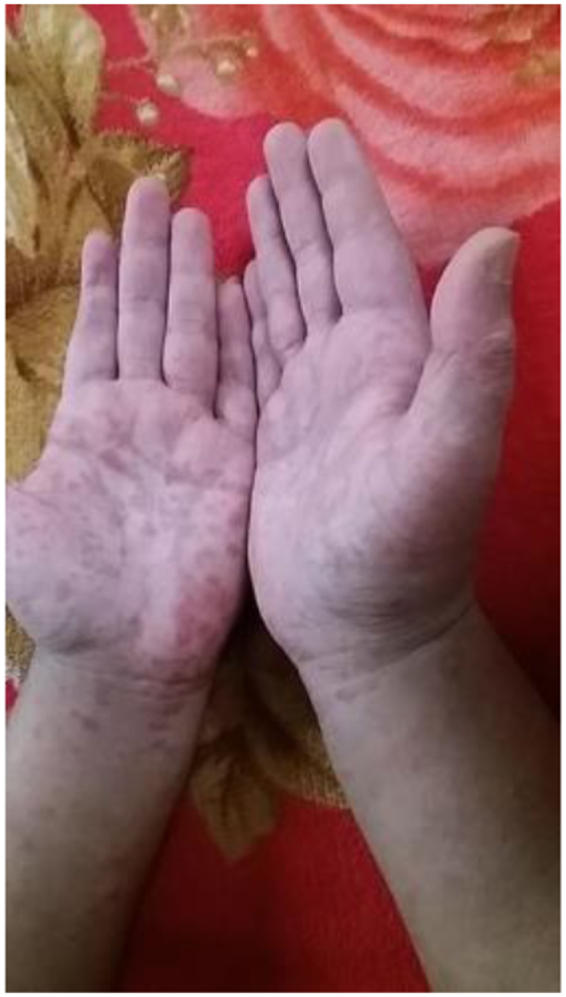
Multiple reddish-purple spots and papules on the palms and fingers.

**Figure 5.**
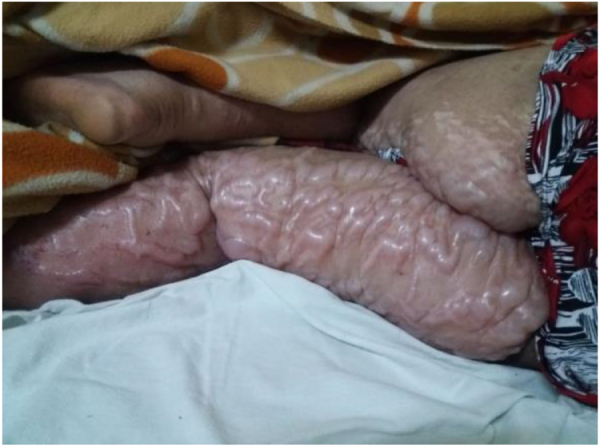
Leg edema with a stretched, shiny, and “peau d’orange” textured skin appearance.

**Figure 6.**
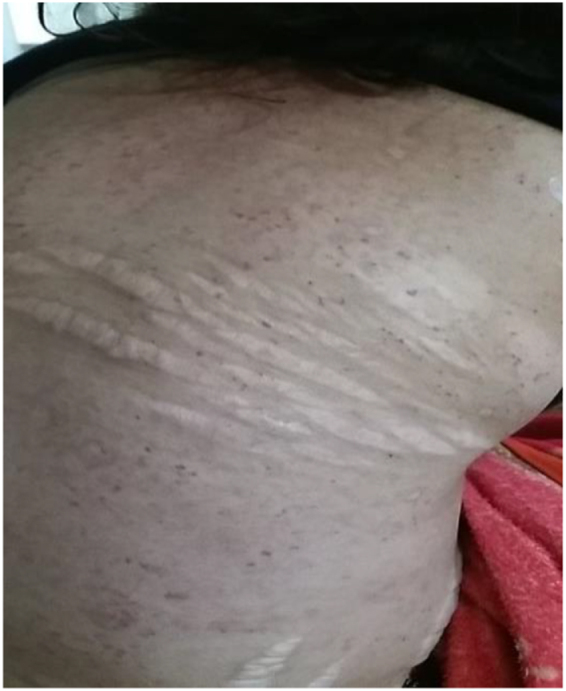
Multiple white linear stretch marks (striae distensae) with diffuse small brownish spots on the trunk of a patient.

**Figure 7.**
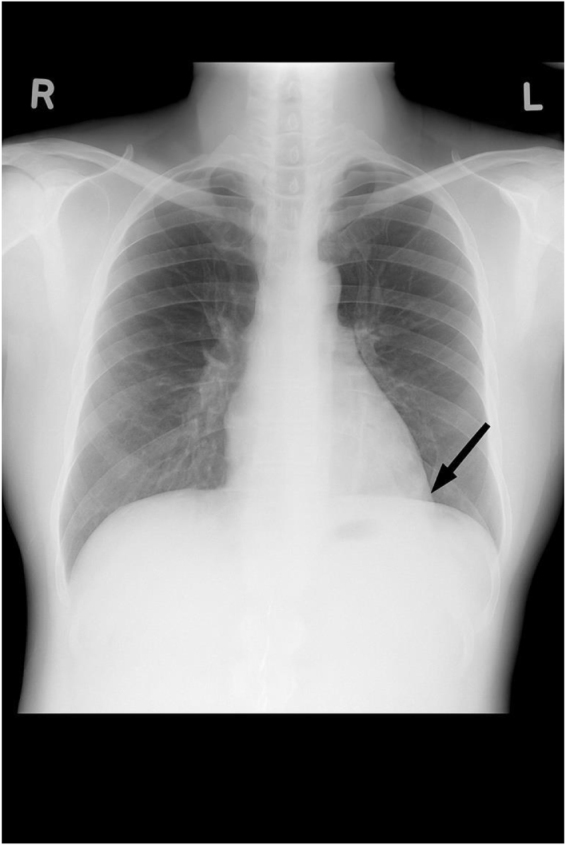
X-ray shows blunting of the right costophrenic angle but no parenchymal infiltrates or cavitary lesions were present.

**Figure 8.**
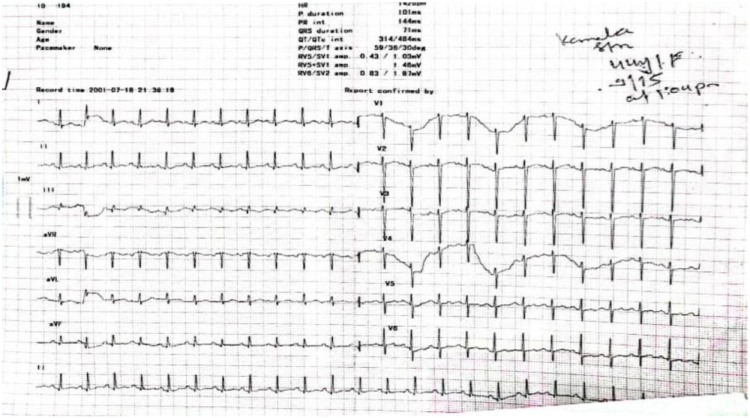
ECG shows normal sinus rhythm.

On examination, the patient appeared pale but had no lymphadenopathy or organomegaly. Cutaneous examination revealed erythematous plaques with pustules and crusting over the right cheek and jawline (Fig. 1), along with multiple erythematous, scaly, and hyperpigmented plaques with lichenification over the back (Fig. 2). A rash consisting of red-brown macules with areas of hypopigmentation was noted over the arm and shoulder (Fig. 3). Multiple reddish purple macules and papules were present on the palms and fingers (Fig. 4). Bilateral lower-limb edema with stretched, shiny skin and a *peau d’orange* appearance was observed (Fig. 5). Multiple white linear striae with diffuse hyperpigmented macules were noted over the trunk (Fig. 6). Abdominal examination revealed mild tenderness. Respiratory examination suggested features of pleural involvement. Chest radiography demonstrated blunting of the right costophrenic angle without parenchymal infiltrates or cavitary lesions (Fig. 7). ECG showed normal sinus rhythm (Fig. 8). Investigations showed anemia, hypoalbuminemia, and hyponatremia. Immunological studies revealed a strongly positive antinuclear antibody (ANA) with a homogeneous pattern and mitotic spindle enhancement. The autoantibody profile showed positivity for anti-dsDNA, anti-Smith, U1-RNP, SSA (Ro), SSB (La), Ro-52, histone, and nucleosome antibodies. Rheumatoid factor was also positive, while KU antibody and DFS70 were borderline positive. Serum creatine phosphokinase was within normal limits.

Based on the constellation of long-standing inflammatory polyarthritis, pleuritic chest pain, constitutional symptoms, characteristic autoantibody profile, and a strong family history, the most likely diagnosis was SLE with pleural involvement and polyarthritis. Differential diagnoses considered included rheumatoid arthritis with extra-articular manifestations, mixed connective tissue disease, and tuberculosis. The patient was started on systemic corticosteroids (prednisolone at an initial dose of 1 mg/kg/day) to rapidly control active inflammation and pleural involvement. Hydroxychloroquine was initiated for its disease-modifying and protective cardiovascular and renal effects. Due to the severity and chronicity of her arthritis and pleural disease, a steroid-sparing immunosuppressant, azathioprine, was considered and gradually introduced. Supportive management included analgesics for pleuritic pain, proton pump inhibitors for gastric protection, and calcium plus vitamin D supplementation to reduce steroid-related bone loss. She received nutritional counseling to address hypoalbuminemia and improve overall nutritional status. Regular monitoring of blood counts, renal and liver function, and complement levels was planned to assess disease activity and treatment toxicity. At the time of presentation, the patient did not have clinical or laboratory features suggestive of lupus nephritis, such as haematuria, proteinuria, or elevated serum creatinine. Hence, renal biopsy was not indicated in this case. However, the patient was counseled and will continue to be monitored for any future renal involvement. Family counseling was provided regarding the familial nature of SLE, potential disease flares, and the importance of adherence to therapy and regular follow-up.

## Discussion

SLE is a chronic autoimmune disorder, characterized by production of autoantibodies directed against nuclear and cytoplasmic antigens, which may affect several different organs, with a plethora of different clinical and immunologic abnormalities, characterized by a relapsing and remitting clinical course^[6]^. Several genetic, immunological, endocrine, and environmental factors play a role in the etiopathogenesis of SLE^[7]^. Low-density neutrophils (LDNs) and platelet-expressed Toll-like receptor 7 (TLR7) play key roles in SLE pathogenesis, through NETosis activation. Another potential driver of pathogenesis may be the PBX1/STAT3 axis and XIST/FOS axes^[8]^. Primary respiratory disease occurs in about 25–30% of patients with lupus during the course of the disease. Apart from pleural disease that occurs in 21% of patients, the other respiratory manifestations are uncommon, occurring in less than 4% of cases. Of the lung parenchymal manifestations, the most commonly observed was pneumonitis (3.6%)^[9]^. In our case, patient had a history of chest pain, aggravating during inspiration, fever with chills, rigor, and night sweats signifying pleural involvement.

Arthritis is a common manifestation in SLE, observed in up to 90% of patients. It often occurs at disease onset and is part of the classification criteria, but it is usually non-erosive^[10]^. Similar was seen in our patient, where a long-standing arthritis was there. In the general population, the risk of developing SLE is approximately 0.1%, while for females, it is around 0.2%. On average, about 7% of SLE patients have first-degree relatives with the same disease^[11]^. The risk of first-degree relatives ranges from 4% to 8%, but in some cases, it can be higher, with sisters of SLE patients having a risk of up to 10%^[12]^.

Our patient had a positive family history in both her sister and her mother. SLE diagnosis is based on a combination of clinical features and laboratory tests, guided by EULAR/ACR classification criteria. After meeting the entry criterion of positive ANA test, weighted clinical and immunologic criteria are scored, where a total score of 10 points or more, including at least one clinical criterion, classifies a patient as having SLE, which was true in our case as well^[13]^. Treatment is individualized, ranging from antimalarials and NSAIDs for mild disease to immunosuppressants and biologics for severe organ involvement^[14]^. Prednisolone, hydroxychloroquine, and azathioprine were used to treat our patient.

## Conclusion

This case highlights the multisystem nature of SLE and the need for high clinical suspicion in patients presenting with inflammatory polyarthritis and unexplained pleural effusion. Early diagnosis and immunosuppressive therapy can significantly improve outcomes and prevent long-term complications. The presence of a strong family history in this case emphasizes the genetic predisposition associated with SLE. Clinicians should be vigilant in screening first-degree relatives presenting with autoimmune symptoms.

## Data Availability

The data that support the findings of this study are available from the corresponding author upon reasonable request.
